# A Chemical Potential Equation for Modeling Triboelectrochemical Reactions on Solid–Liquid Interfaces

**DOI:** 10.3389/fchem.2021.650880

**Published:** 2021-04-23

**Authors:** Chenxu Liu, Yu Tian, Yonggang Meng

**Affiliations:** State Key Laboratory of Tribology, Tsinghua University, Beijing, China

**Keywords:** triboeletrochemistry, adsorption/desorption, solid–liquid interface, boundary lubrication, potential control

## Abstract

Triboelectrochemical reactions occur on solid–liquid interfaces in wide range of applications when an electric field strong enough and a frictional stress high enough are simultaneously imposed on the interfaces. A characteristic of triboelectrochemical reactions is that not only the thermal energy but also the electrical and mechanical energies can activate, assist, or mitigate the solid–liquid interface chemical reactions, the products of which affect electrical and tribological behavior of the interfaces inversely. In previous studies, we have found that the coupling of frictional and electric effects could physically change the migration, adsorption, and desorption behaviors of the polar molecules, ions, or charged particles included in aqueous or nonaqueous base lubricant toward or away from the interfaces and thus control the boundary lubrication. Recently, we have found that the friction coefficient and surface appearance of some kinds of metals could also be modulated to some extent even in pure water or pure base oils under external electric stimulations. We attribute these changes to the triboelectrochemical reactions occurred when a strong external electric field is imposed on. Based on the effective collision model of chemical reactions, a chemical potential equation, which includes both electrical and mechanical contributions, has been derived. The proposed chemical potential equation can be used to explain the observed triboelectrochemical phenomenon in experiments. Based on the model, a novel method for oxidation coloring of the selected areas in metal surfaces is proposed. Together with the physical adsorption and desorption model of lubricant additives, the triboelectrochemical reaction model can well explain the phenomena of potential-controlled boundary lubrication in different lubrication systems and also provides a theoretical basis for other solid–liquid interface processes under the effects of electromechanical coupling.

## Introduction

Drilling wood to make fire is well known as the beginning event of mankind civilization. From the viewpoint of modern science, making fire through drilling wood is a typical tribochemical process. In modern technology, tribochemistry has been greatly studied and intentionally applied to many industrial practices (Hsu et al., 2002; Yu et al., 2012). For example, through the tribochemical reactions of lubricating additives at friction interfaces, zinc dialkyl dithiophosphate (ZDDP) or molybdenum dithiophosphate (MoDDP) is widely used to improve wear resistance of materials (Spikes, 2015; Zhang and Spikes, 2016). In the field of integrated circuit manufacturing, chemical mechanical polishing (CMP) is used as an effective surface planarization method (Cadien and Nolan, 2018). Meanwhile, interdisciplinary studies between tribochemistry and electrochemistry have also been done a lot. As early as in 1950s, Bowden and Young had introduced the potentiostatic technique of electrochemistry to change the coefficient of friction (COF) during the sliding process (Cadien and Nolan, 2018). In 1990s, electrochemical workstations were used to study the tribochemical mechanism of ZDDP (Wang et al., 1989; Tung and Wang, 1991; Wang and Tung, 1991). In the past two decades, great efforts have been devoted to control the tribological performance of various tribosystems through an external electric field, and such active approaches are regarded as potential-controlled friction or potential-controlled boundary lubrication (Meng et al., 2001; He et al., 2010).

**FIGURE 1:**
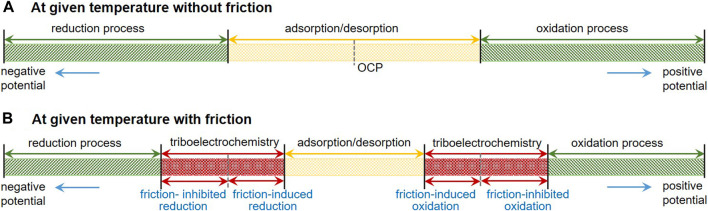
Schematic diagram of the electrode processes at a given temperature without **(A)** and with **(B)** friction.

In relatively mild electric field conditions, the potential-induced physical changes are reversible adsorption/desorption and morphology transition of responsive constituents of cations, anions, and/or charged nanoparticles dissolved or dispersed in solutions (He et al., 2011; Yang et al., 2014; Liu et al., 2018; Liu et al., 2019; Meng and Liu, 2020) and phase transformation of polar molecules like water as well as electrical double layer (EDL) interactions (Pashazanusi et al., 2017; Li et al., 2018). For strong electric field conditions, electrochemical reactions, such as electrolysis of the solution, are involved, resulting in irreversible changes of oxidation/reduction and tribofilm formation/resolving on the electrodes as well as decomposition of the solution (Chang et al., 2002). Figure 1 presents a schematic diagram of the electrode processes at a given temperature without and with friction. The traditional electrochemical processes without the influence of friction generally include adsorption/desorption process when the applied surface potential is within the thermodynamic potential window (–0.7 V to +1.0 V vs. saturated calomel electrode (SCE), for Pt electrode in buffer aqueous, pH = 7; OCP means open circuit potential) (Izutsu, 2009), reduction process at negative potentials and oxidation process at positive potentials. While for the electrode processes with the influence of the friction, triboelectrochemical processes including friction-induced reduction and friction-inhibited reduction and friction-induced oxidation and friction-inhibited oxidation should be involved as shown in Figure 1B. However, when the lubricant contains ionic surfactants and/or charged nanoparticles, which are called the electrostatic responsive components, the adsorption or desorption behaviors of these additives play an important role on the potential-controlled boundary lubrication process.

Owing that the electrochemical reaction process involves electrochemical corrosion, most of previous researches focuses on the adsorption/desorption process to realize the active control of boundary lubrication. In fact, no matter what liquid is used as the lubricating medium, or no matter what mechanism dominates friction and wear in the tribosystem, there are always two distinct zones on the liquid-electrified solid interfaces, one is inside the frictional contact area and the other is outside the frictional contact area. The electrochemical behavior is different in the two zones when an imposed electric potential is present on the solid surface. In this work, a model of triboelectrochemical reaction which includes both electrical and mechanical contributions is proposed based on the effective collision model of chemical reactions. Different from previous studies, the triboelectrochemical model emphasizes the difference of electrochemical reactions between the frictional contact zone and noncontact zone. It can well explain the observed phenomena of potential-controlled boundary lubrication for pure liquids and the triboelectrochemical reaction products in friction contact area. Most importantly, the model is expected to provide a theoretical basis for many other solid–liquid interface processes under the effects of electromechanical coupling, such as the scratching of the surface under electrochemical conditions (Wang and Li, 2005), the highly efficient electrochemical mechanical polishing (Joo and Liang, 2013; Li et al., 2013), the rolling current-carrying contact in wet environment (Sun et al., 2019; Sun et al., 2020), and so on.

## Chemical Potential Model for Triboelectrochemical Reactions

To explore the mechanism of triboelectrochemical processes, the basic knowledge on electrode process, as well as sliding friction process, should be considered simultaneously. The thermodynamic model of chemical reactions including the collision model and transition state theory shows some possible relationship between the electrochemistry and mechanochemistry in microscopic mechanism. Hence, our analysis and discussion will start with the basic thermodynamic model of chemical reactions.

### Thermodynamic Model for Chemical Reactions

**FIGURE 2:**
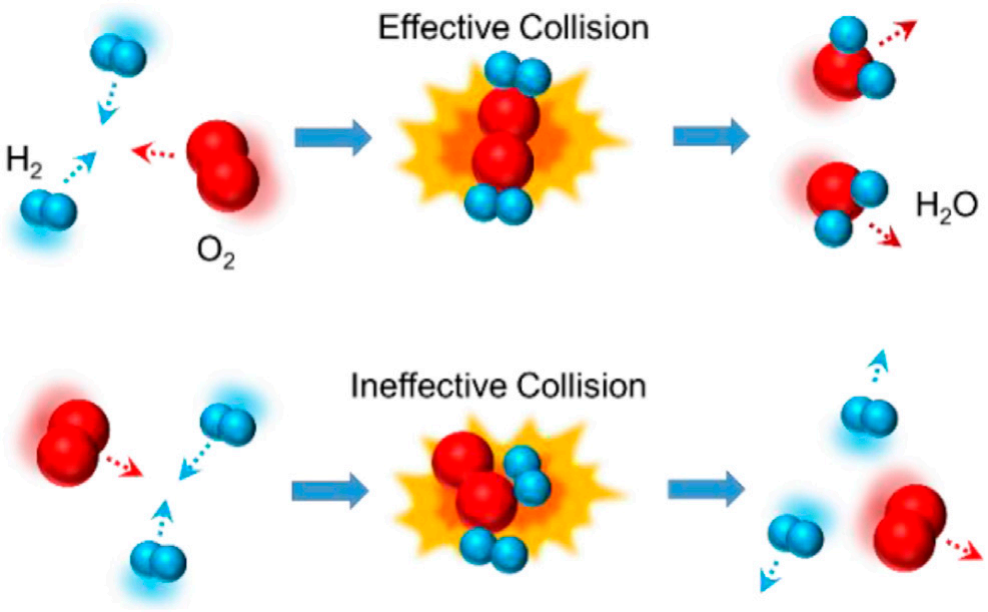
Schematic diagram of the collision model for reactions between H_2_ and O_2_.

Chemical reaction is defined as a process in which one or more substances, the reactants, are converted to one or more different substances, the products. It implies the changes in the interactions between particles, including atoms, molecules, or ions. Since the 19th century, the understanding of chemical reactions involves kinetic molecular theory, collision theory, and transition state theory (Pechukas, 1981). Figure 2 shows the models of chemical reaction and physical reaction between hydrogen and oxygen, which are also named as effective collision and ineffective collision.

According to the collision theory and transition state theory, the molecular species having the maximum energy is called the activated complex, and its state is called the transition state. The factors affecting the effective collision between particles include temperature (related to the kinetic energy of particles), substance concentration in solution or pressure/partial pressure of gas (related to the number density of particles), and so on. For a solid, its reaction rate is mainly related to its specific surface area or surface energy. For example, the reaction efficiency of nanomaterial is generally higher than that of the same bulk material. In addition, it also depends on the location of particle collisions, so researchers can choose appropriate catalysts to increase or inhibit the reaction rate or introduce some active sites to change the reaction processes.

The above collision model gives a qualitative explanation of chemical reaction, while the direction and rate of chemical reactions are still not explicitly described. In 1874, Willard Gibbs, a great American physicist and chemist, introduced a symbolism, in which the coefficient of form (the partial molar Gibbs energy) is called the chemical potential (μ), and used it to deal with the matter equilibrium of multicomponent and multiphase systems, namely, chemical equilibrium and phase equilibrium. His application of thermodynamic theory converted a large part of physical chemistry from an empirical into a deductive science (Junji et al., 2009).

Up to now, thermodynamic methods for dealing with chemical equilibria have been generally accepted. The basic idea is to establish a relationship between the chemical potential and the Gibbs function of a chemical reaction, like shown in Eq. 1.

$$\Delta_{r}G_{m}=\sum_{B}v_{B}\mu_{B},$$ (1)

where B is for any of the components in a reaction, $\Delta_{r}G_{m}$ is the standard molar Gibbs free energy, $\mu_{B}$ is the chemical potential of component B, and $v_{B}$ is the stoichiometric number of component B ($v_{B}$ is positive for products and negative for reactants).

According to the Gibbs free energy criterion, when the reaction reaches equilibrium under constant temperature and constant pressure, $\Delta_{r}G_{m}$ equals to 0. Hence, Eq. 2 can be obtained according to isothermal equation.

$$\Delta_{r}G_{m}^{\theta }=-\mathrm{RT\times ln}K^{\theta },$$ (2)

where $K^{\theta }$ is the dimensionless equilibrium constant of the chemical equilibrium. $\Delta_{r}G_{m}^{\theta }$ is the standard molar Gibbs free energy, which can be obtained from the basic thermodynamic data. Hence, the composition of the system at chemical equilibrium can be calculated theoretically with $K^{\theta }$. Temperature can change the composition of the equilibrium by changing $K^{\theta }$, even the direction of the reaction. For the reactions with ${\sum v_{B}\neq 0}^{\text{​}}$, some other factors besides temperature, such as pressure, inert gas, and ratio of reactants, which cannot change the equilibrium constant but can shift the reaction equilibrium, will also affect the reaction rate (Junji et al., 2009).

### Thermodynamic Model for Electrochemical Reactions

According to the above theoretical models for chemical reactions, the effects of some physical and chemical factors on chemical reactions can be studied by considering temperature, gas pressure, contact of reactants and products, and so on. Among them, the influence of electric field on chemical reactions is widely researched.

According to the second law of thermodynamics, the Gibbs free energy change ΔG of the system at constant temperature and pressure equals to the reversible non-volume work exchanged between the system and the environment. For ΔG<0, electrochemical reactions occur spontaneously, resulting in electrical energy doing work externally. For ΔG>0, an external input of energy (in this case, electricity) is needed to cause chemical reactions to occur (Junji et al., 2009). The above two types are commonly referred to as galvanic cell and electrolytic cell. This work focuses on the latter, the effect of external electrical energy on chemical reactions.

As we know, electrochemical reaction is a process of oxidation and reduction. That is to say, some chemical substances gain electrons and others lose electrons. The electrochemical process is equivalent to increasing the number density of active particles in effective collisions. Therefore, the catalytic effect of the applied electric field on the redox reaction lies in the active supply of electrons or holes, which shortens the energy or process required by the original reaction. Take electrolysis of water, for example, H^+^ ions react by gaining electrons and H_2_ molecules are formed at the cathode, while O^2−^ ions (in H_2_O molecules) lose electrons at the anode and are oxidized.

Of course, electric field can not only change the direction of a chemical reaction, for example, it is almost impossible for water to spontaneously produce H_2_ and O_2_ at room temperature and pressure without an electric field but also influence the reaction rate. Many experimental results show that the reaction rate can be changed by orders of magnitude by simply changing the electrode potential, with other conditions unchanged. Hence, the way in which electrode potential affects chemical reactions can be summarized as follows. Electrode potential changes the surface concentration of some particles, indirectly affecting the reaction rate of the rate-determining step in which these particles participate. Second, electrode potential may directly change the process of the electron transfer and then the rate of the whole electrode reaction. The former refers to the physical process of mass transfer or adsorption/desorption, while the latter refers to the electrochemical reaction process.

The influence of electrode potential on the reaction rate of electrochemical process is mainly realized by changing the reaction activation energy. If the electrode potential changes Δφ, the activation energies of the anodic and cathodic reactions can be presented as Eqs 3, 4, respectively (Zha, 2002).

$$E_{a}=E_{a}^{0}-\mathrm{\beta zF\Delta}\varphi ,$$ (3)

$$E_{c}=E_{c}^{0}\mathrm{+\alpha zF\Delta}\varphi ,$$ (4)

where $E_{a}^{0}$ and $E_{c}^{0}$ are the activation energies of anodic and cathodic reactions, respectively, β and α are the transfer coefficient of applied potential alters activation energy for oxidation and for reduction, respectively, *F* is the Faraday’s constant, and *z* is the reactive charge number.

According to the collision model and transition state theory, combining with the above analysis, new substances (products) can be formed, when the activation energy reaches the energy required for activated complex in the transition state. Otherwise, just the electromigration or adsorption/desorption can occur on the interface between solid and liquid, instead of chemical reactions.

### Mechanochemical Reactions and Thermal Activation Models

Besides electrochemistry, another term we focused on in this work is tribochemistry. As we know, tribochemistry belongs to the mechanochemistry. Hence, the development and theory models of mechanochemistry are shortly discussed before that of the tribochemistry. Actually, comparing with the electrochemistry, the development of mechanochemistry is relatively immature, although it has a long history (Takacs, 2013). The terminology of mechanochemistry was coined by Ostwald, a German scientist, in his publications. In the 19th century, Parker published the first picture of mechanochemical reactor. He drew attention on the importance of controlling the atmosphere during milling process (Baláž, 2008). Later, using this idea, scientists prepared some small size materials, which are called nanoparticles today.

With the development of the new materials and advanced testing equipment, mechanical destruction of the chain structure or interaction between groups of organic molecules has been studied in recent years. Single-molecule mechanochemistry of macromolecules was measured by atomic force microscope (Zhang and Zhang, 2003). In addition, the formation of the ZDDP tribofilms (Cao and Meng, 2018) and synthesis of some kinds of mechanoluminescent also belongs to the field of the mechanochemistry. Another phenomenon is that friction could cause a phase transition for some solid materials. For example, in our previous study (Liu et al., 2020), the phase transition of some MoS_2_ additives from hexagonal to rhombohedral had been observed, when they were dispersed in ester oil and worked under the state of the boundary lubrication.

There are two basic mathematic models to describe mechanochemistry. The first one can be referred as one-dimensional (1D) model because only normal stress is considered (Eyring, 1935; Wynne Jones and Eyring, 1935; Eyring, 1936; Kauzmann and Eyring, 1940), while the second one is a two-dimensional (2D) model (Briscoe and Evans, 1982; Umer et al., 2020) because both compression stress and shear stress are considered. Actually, both models belong to Eyring model, which can also be considered as a type of transition state theory. In 1935, Eyring and his co-workers proposed some equations, based on transition state theory, to calculate the absolute rates in chemical reactions (Eyring, 1935; Wynne Jones and Eyring, 1935). In 1936, they developed the model to explain the results of viscosity, plasticity, and diffusion (Eyring, 1936). For example, they gave the equation to present the relationship of the viscosity and the temperature as well as shear stress. In 1940, they used the model to explain the viscous flow of large molecules and thought that the potential function for the bond could be given by a Morse potential function in Eq. 5 (Kauzmann and Eyring, 1940).

$$V\left(r\right)=D{\left(1-e^{-a\left(r-r_{0}\right)}\right)}^{2},$$ (5)

where r is the length of the bond, $r_{0}$ is the equilibrium separation of the atoms, D is the dissociation energy, and a is related with D and k, as shown in Eq. 6.

$$\mathrm{a=}\sqrt{k/\mathrm{2D}},$$ (6)

where k is the force constant of the bond in the neighborhood of the equilibrium separation.

**FIGURE 3:**
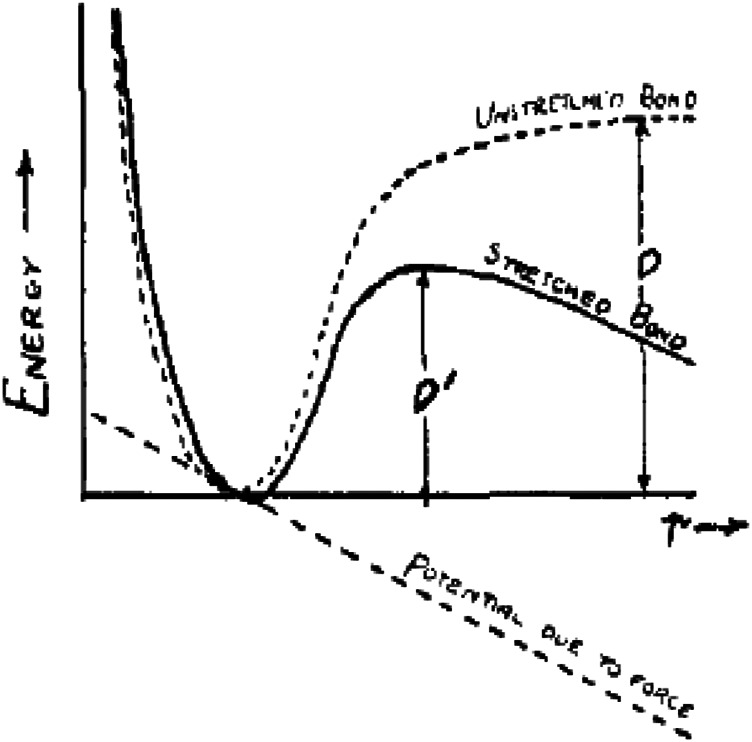
Potential function for the bond given by a Morse function. When the bond is under tension due to a constant force *f* pulling on either end, the activation energy required to break the bond is diminished from *D* to *D′* (Kauzmann and Eyring, 1940). (X-coordinate stands for the length of the bond, *r*, while the Y-coordinate stands for “energy.” The dotted and solid curves represent “unstretched bond” and “stretched bond,” respectively. The dotted straight line represents “potential due to force”).

To break a bond under no external vibration, an energy *D* must be supplied, like shown in Figure 3. However, if the bond is under the tension (f), the activation energy required to break the bond will be diminished from *D* to *D′*.

Moreover, they concluded the relationship between *D* and *D′* in Eq. 7.

$$\frac{D^{\prime }}{D}=x\log\left(\frac{1-x-\sqrt{1-2x}}{x}\right)+\sqrt{1-2x},$$ (7)

where x=f/Da.

In 2004, Bustamante et al. studied the effect of force on the free energy of a two-state system for mechanical processes in biochemistry (Bustamante et al., 2004). The difference between their model and the model in Figure 3 is that the possible shifts in position for the reactant (A) and product (B) are considered. The free energy change of states A and B upon stretching is named as $\Delta G_{\mathrm{stretch}}^{A\to B}\left(f\right)$ in Eq. 8.

$$\Delta G^{0}-F\Delta x+\Delta G_{\mathrm{stretch}}^{A\to B}\left(f\right)=-k_{B}T\cdot \ln K_{eq}\left(f\right),$$ (8)

where $\Delta G^{0}$ is the standard state free energy, Δx is the shift in position for the reactant A and product B, $k_{B}$ is Boltzmann constant, T is temperature, and $K_{eq}\left(f\right)$ is the equilibrium constant with the external force. Actually, the relative shift of A and B is generally small. Hence, the free energy change of states A and B upon stretching can be ignored ($\Delta G_{\mathrm{stretch}}^{A\to B}\left(f\right)=0$) under common conditions, and the change of the potential energy is only fΔx.

In 2006, Wiita et al. used single-molecule technique to study the force-dependent chemical kinetics of disulfide bond reduction (Wiita et al., 2009). Also, they thought that the extra force could change the potential curve. They predicted that the transition state was located at 0.34 A along the linear reaction coordinate. Most importantly, they calculated that applying 400 pN of force reduced the activation energy barrier by 8.2 kJ/mol.

In summary of the above research studies, it can be concluded that after applying of a force *f,* the free energy will be decreased by fΔx. The force will possibly lead to the relative shift in position for the reactant and product. For most situations, the relative shift is very small and can be ignored. Moreover, Eq. 8 indicates that the equilibrium constant $K_{eq}\left(f\right)$ depends on the applied force. By applying a force assisting (*f* > 0) or opposing (*f* < 0) the transition, the equilibrium of the reaction can be altered.

**FIGURE 4:**
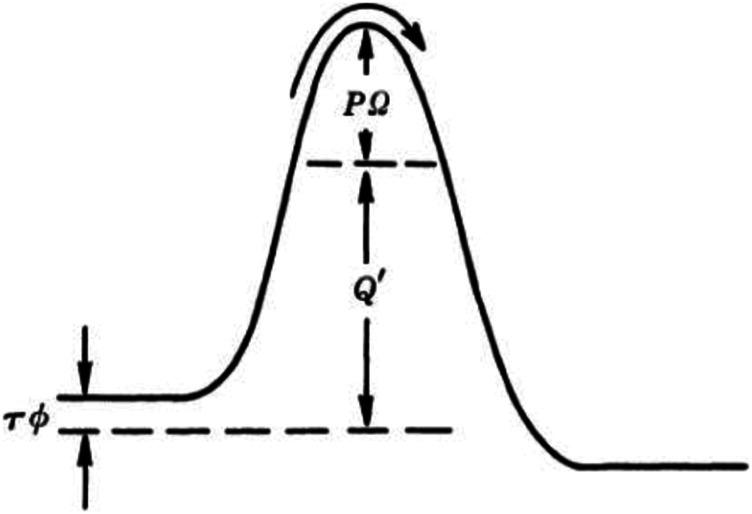
Potential barrier in the thermal activation model. The barrier is modified by the influence of pressure *P* and shear stress τ (Briscoe and Evans, 1982).

In the above mechanochemical models, the direction of the force is one-dimensional. However, for the tribochemistry, there are some different conditions, owing to the presence of pressure and shear force. In 1982, Briscoe proposed a model to explain the sliding friction process between the Langmuir–Blodgett carboxylic acid layers (Briscoe and Evans, 1982). Figure 4 shows the potential barrier in the thermal activation model. The barrier is present due to the interactions with the neighboring molecules. All molecules need to overcome this energy barrier for any dislocation and subsequent continuous motion.

In their model, the contact pressure (*P*) increases the barriers potential, but shear stress (τ) reduces the effort required for relative sliding of contacting solids. It is worth mentioning that the equation was from and verified by experiments. The Eyring shear stress associated with energy barrier components can be described by

$$\tau \phi =k_{B}T\ln\left(\frac{v}{v_{0}}\right)+\left(Q+P\Omega \right),$$ (9)

where *Q* is the activation energy to initiate sliding. *Ω* and ϕ are pressure and shear activation volumes, respectively. $k_{B}$ is the Boltzmann constant, *T* is the operating temperature, *v* is the sliding velocity, and *v*_*0*_ is a characteristic constant velocity with a value of 20 m/s, which is estimated from the product of molecules vibrational frequency (about 10^11^ s^−1^) and the lattice constant (0.2 nm) (Briscoe and Evans, 1982).

Comparing the above discussions, although the force directions of the 1D model in Figure 3 and the 2D model in Figure 4 are different, both of them belong to Eyring model and based on the transition state theory, which indicates some potential connection with the thermodynamic model for electrochemical reactions.

### Triboelectrochemical Potential

According to the above analysis, the collision model and transition state theory are accepted to discuss the influence of electricity on chemical reactions, as well as the pressure or shear force on the sliding process. In fact, most of these models are based on the Gibbs free energy. It was generally assumed that the decrease of activation energy or potential barrier during the thermal activation is linearly related to tensile stress. However, M. Gutman proposed that chemical potential should play a vital role in mechanochemistry processes of metals in 1974 (Gutman et al., 1989). He pointed out that the Gibbs free energy approach had some obvious disadvantages. For example, when compressive stress replaced the tensile stress, it was considered that the pressure would retard the reaction, so that the chemical equilibrium would transfer toward the bond recombination direction. It was obviously not consistent with the known instances in which pressure could accelerate solid–solid reactions. Therefore, Gutman proposed that when considering the effect of forces on chemical reactions, it was not the energy barrier that needed to be considered but the chemical potentials involved in the mechanical action of the reaction components (reactants, activated complexes, and products). The sign of the effect depended on whether the mechanical stress impeded or promoted the change in the volume of the system during the reaction (Gutman et al., 1989).

In recent years, Chen indicated that all of the thermodynamic properties of a material at a given temperature and pressure can be obtained from knowledge of its chemical potential (Chen, 2019). Whether the stability of substances, such as chemical species, compounds, and solutions, or their tendency to chemically react to form new substances, to transform to new physical states, or to migrate from one spatial location to another, a difference in chemical potential between two locations or a chemical potential gradient is the driving force. Therefore, Chen suggested that when thermodynamics was applied to material science and engineering, the concept of chemical potential should be adopted in most cases, rather than the concept of Gibbs free energy.

As mentioned above, chemical potential (*μ*) is considered to determine the stability of substances, and their tendency to chemically react to form new substances, to transform to new physical states, or to migrate from one spatial location to another (Chen, 2019). If *n* mol of A is present in a mixture of A, X, etc., the chemical potential *μ* of A is defined as the partial molar Gibbs free energy (*G*), as shown in Eq. 10 (Laidler and Meiser, 1999).

$$\mu ={\left(\frac{\partial G}{\partial n}\right)}_{T,\;P,n_{X}},$$ (10)

where *n* is the number of particles of species A, *n*_X_ the number of substances with the exception of A, *P* denotes the pressure, and *T* denotes the temperature.

According to the molar Gibbs free energy, the chemical potential of any component A in the mixture in real condition (nonideal system) can be expressed in Eq. 11.

$$\mu =\mu^{\theta }+RT\cdot \ln a,$$ (11)

where $\mu^{\theta }$ is the chemical potential of a mole of substance A at standard pressure (101.325 kPa), R is the gas constant, with the value of 8.314 J mol^−1^ K^−1^, and a is the effective concentration of the A, also named as activity (Laidler and Meiser, 1999).

For an electrode system, when T, *P*, and *n*_X_ of all components other than A itself are held constant, a change of the particle number of A not only changes the chemical potential but also influences the electric potential energy due to the introduction of the charge. The electrochemical potential $\mu_{E}$ can be divided into the work (W_1_) required for the transfer of particle in the homogeneous volume and the work (W_2_) required for transfer to point outside to inside the shell in the real phase (Zha, 2002). Hence, the electrochemical potential can be expressed as Eq. 12.

$$\mu_{E}=\mu +zF\varphi ,$$ (12)

where *z* is the ionic valence number of A, *F* is the Faraday’s constant, and φ is the potential of the system.

Combining Eqs 11, 12, the electrochemical potential can be expressed in Eq. 13.

$$\mu_{E}=\mu^{\theta }+RT\cdot \ln\left[a\cdot \exp\left(\frac{zF\varphi }{RT}\right)\right],$$ (13)

where a⋅exp(zFφ/RT) is defined as the electrochemical activity, named as $a_{E}$.

According to the thermodynamic laws, for condensed matter, its volume (*V*) varied little with pressure (*P*). Here, *V* is considered as a constant. When the matter is subjected to an excess pressure (*∆p*), its chemical potential, also named as mechanochemical potential, can be expressed in Eq. 14.

$$\mu_{P}=\mu +V\Delta p.$$ (14)

Combining the Eqs 11, 14, the mechanochemical potential can be given by

$$\mu_{P}=\mu^{\theta }+RT\cdot \ln\left[a\cdot \exp\left(\frac{V\Delta p}{RT}\right)\right],$$ (15)

where a⋅exp(VΔp/RT) is defined as the mechanochemical activity, named as $a_{P}$ (Gutman et al., 1989).

As friction process involves both pressure and shear force, tribochemical potential can be expressed similarly in Eq. 16.

$$\mu_{f}=\mu^{\theta }+RT\cdot \ln a+F_{P}l_{⊥}+F_{\tau }l_{∥},$$ (16)

where $\;F_{P}$ and $F_{\tau }$ present the value of the pressure and shear force, respectively. $l_{⊥}$ and $l_{∥}$ present the displacement in the direction of pressure and shear force, respectively, whose sign depends on whether it impedes or promotes the reaction during the changing stage. Inspired by the thermal activation model, the works done by pressure and shear force can be presented by the pressure activation energy (PΩ) and shear force activation energy (τϕ). Then, the expression of the tribochemical potential can be expressed as follows.

$$\mu_{f}=\mu^{\theta }+RT\cdot \ln a+P\Omega +\tau \phi ,$$ (17)

$$\mu_{f}=\mu^{\theta }+RT\cdot \ln\left[a\cdot \exp\left(\frac{P\Omega +\tau \phi }{RT}\right)\right],$$ (18)

where P is the contact pressure and τ is the shear stress. Ω and ϕ are pressure and shear activation volumes, respectively. a⋅exp(PΩ+τϕ/RT) is defined as the tribochemical activity, named as $a_{f}$.

According to the additivity of extensive quantities, as well as the independent property of force and electricity, the triboelectrochemical potential can be defined and presented in Eq. 19.

$$\mu_{f\&E}=\mu +\left(P\Omega +\tau \phi \right)+zF\varphi .$$ (19)

Combining the Eqs 11, 19, the triboelectrochemical potential can be finally expressed by

$$\mu_{f\&E}=\mu^{\theta }+RT\cdot \ln\left[a\cdot \exp\left(\frac{P\Omega +\tau \phi +zF\varphi }{RT}\right)\right],$$ (20)

where a⋅exp(PΩ+τϕ+zFφ/RT) is defined as the triboelectrochemical activity, named as $a_{f\&E}$.

When the chemical system in the absence of external force is at equilibrium, the Gibbs energy change (∆G) is 0. For the electrochemical potential at a certain temperature and pressure, its activity at equilibrium is a constant, named *a*_*eq*_, and *φ*_*eq*_ is the equilibrium potential. According to the above analysis, the equilibrium potential in the absence of external force can be shown in Eq. 21.

$$\varphi_{eq}=\frac{RT}{zF}\ln\frac{a_{eq}}{a}.$$ (21)

For the triboelectrochemical potential, its activity is also a constant at equilibrium. As we know, equilibrium concentration is only influenced by the temperature for condensed matter systems and the original concentration of the reactants. ${\varphi^{\prime }}_{eq}$ is the equilibrium potential under the effect of external force.

$${\varphi^{\prime }}_{eq}=\frac{RT}{zF}\ln\frac{a_{eq}}{a}-\frac{P\Omega +\tau \phi }{zF}.$$ (22)

Comparing with Eqs 21, 22, the equilibrium potential will be changed under the influence of force and their difference can be given by

$$\Delta \varphi^{\prime }={\varphi^{\prime }}_{eq}-\varphi_{eq}=-\frac{P\Omega +\tau \phi }{zF}.$$ (23)

Assume that the local external pressure is 1 GPa (here, only pressure is considered and shear force is ignored) and the activation volume is 1 mol of metal atoms, some calculations based on Eq. 23 can be carried out as follows. Let us consider the electrochemical reaction in which Fe changes to Fe^3+^, the equilibrium potential change caused by external forces is about 25 mV. For the electrochemical reaction in which Ti changes to Ti^4+^, the equilibrium potential is about 28 mV. For the electrochemical reaction in which Cu changes to Cu^2+^, the equilibrium potential is about 37 mV. That is to say, potential windows will be changed in different liquids under the external force, as shown in Figure 1. In general, friction can promote the process of the oxidation reaction; hence, the required electrode potential can be decreased as discussed above. However, according to the collision model in Figure 2, in the presence of external forces, the space for the collision may be occupied by other materials (such as friction pair material that does not participate in the reaction), resulting in the inhibition of the reaction. In such a case, the sign of activation volumes in Eq. 23 is negative, and friction-inhibited oxidation in the particular area occurs. As the inverse of the oxidation process, the reduction process with the influence of friction can also be presented in Figure 1B.

The occurrence of the triboelectrochemical reactions must correspond to some macro or micro phenomena. Hence, in order to verify the existence and effect of triboelectrochemical reactions, experiments on the potential-controlled boundary lubrication in various pure liquids are carried out, with friction coefficient and tribofilms mainly characterized in the next section.

### Potential-Controlled Boundary Lubrication Based on the Triboelectrochemistry

**FIGURE 5:**
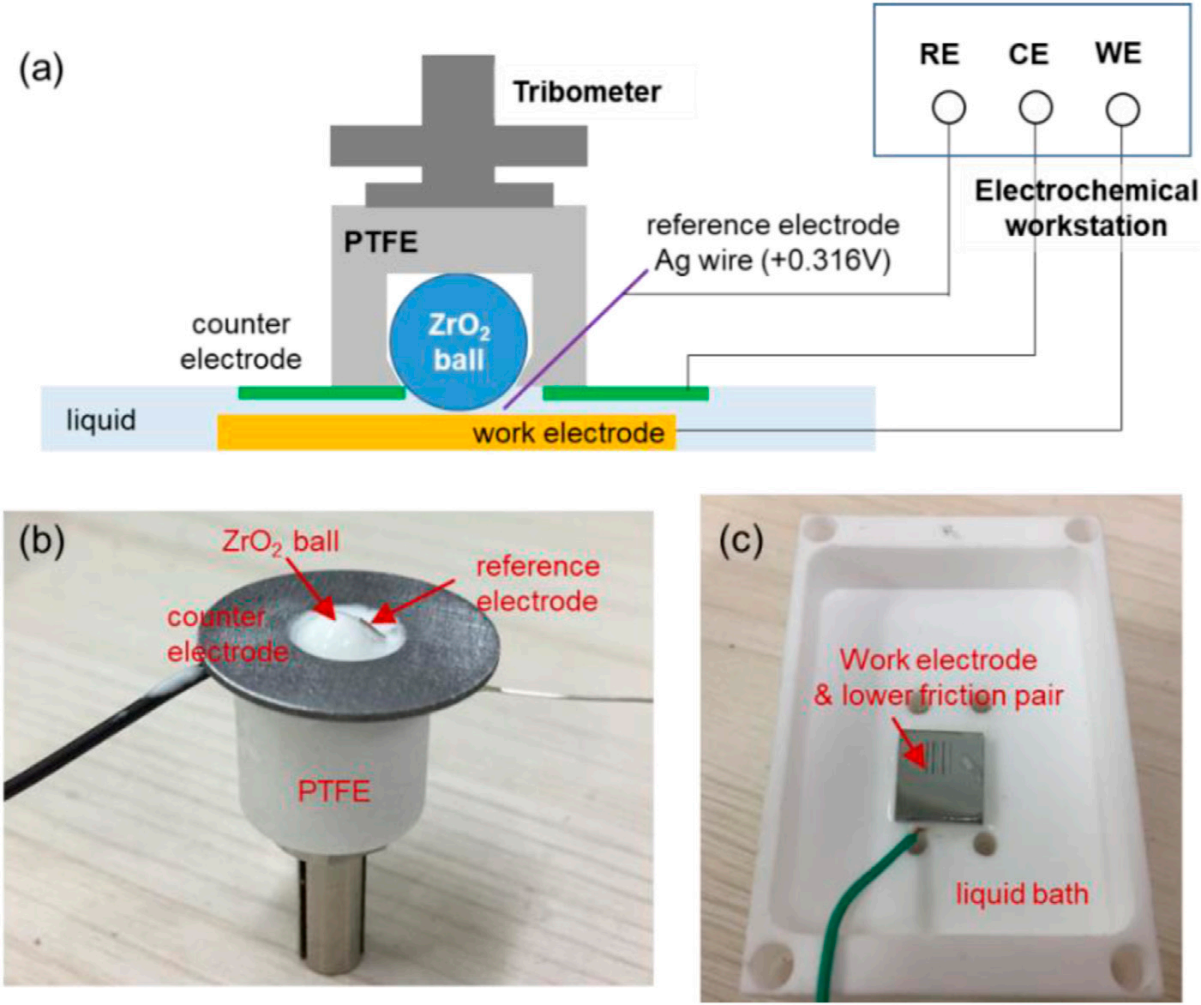
Testing module combining a three-electrode system and a tribometer. **(A)** schematic diagram of the module, **(B)** picture of the upper friction pair section, and **(C)** picture of the lower friction pair section.

Before the verification experiments, a device shown in Figure 5A was designed by combination of a three-electrode system of electrochemical workstation (PGSTAT302N, Auto, Switzerland) and a tribometer (UMT-3, Bruker, Germany). Figure 5B shows the real picture of the upper friction pair section, combining with a counter electrode of Al alloy or steel (inner diameter 5 mm, outer diameter 12 mm, and thickness 1 mm) and a reference electrode of Ag wire (diameter 0.4 mm, electrode potential +0.316 V vs. the standard H electrode). The potential scanning range set in the electrochemical experiment is 0.02 V/s, and the sampling frequency is 50 kHz. To avoid the interference of other factors, all the connection parts in the liquids shown in Figure 5 were sealed with silicone rubber.

In friction tests, Cu plates (20 mm × 20 mm × 20 mm) polished to roughness Ra of about 10 nm were used as a lower friction pair. Zirconium dioxide (ZrO_2_, Ra about 20 nm) balls with a diameter of 6.35 mm were used as an upper friction pair. A micro-tribometer (UMT-3, Bruker, Germany) was used with pure liquids including H_2_O, propylene carbonate (PC), and diethyl succinate (DES) as lubricants. The normal load was set at 3 N and reciprocating frequency at 1 Hz. After the friction tests, morphologies, elemental compositions, and chemical structures on the tracks of the lower friction pair were characterized by using optical microscope (Keyence, Japan), scanning electron microscope (SEM, Quanta 200 Environmental Scanning Electron Microscope, FEI, Netherlands), energy-dispersive spectroscopy (EDS, FEI, Netherlands), and X-ray photoelectron spectroscopy (XPS, MFP-3D-SA, Japan), respectively.

### Potential-Controlled Boundary Lubrication in Pure Water

**FIGURE 6:**
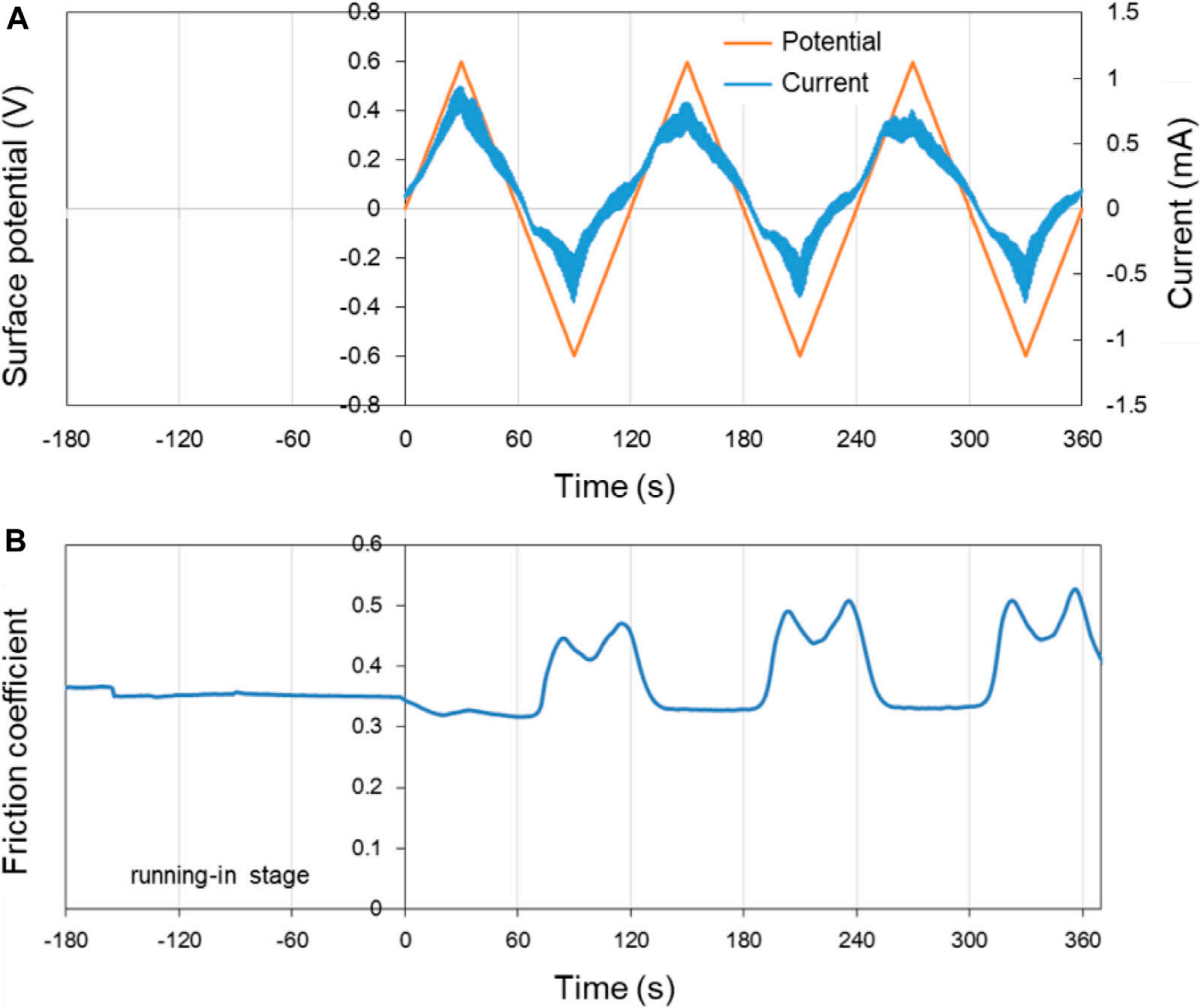
Potential-controlled friction results of the Cu plate vs. ZrO_2_ ball with pure H_2_O as lubricant, with the test range of surface potential ±0.6 V. **(A)** The change of surface potential and current of Cu plate with time and **(B)** the variation curve of friction coefficient with time under the applied surface potentials in **(A)**.

First, pure H_2_O was used as lubricant to explore its potential-controlled boundary lubrication behavior under different surface potentials of the lower friction pair Cu. In the experiment, the surface potential was changed by 0.02 V/s using the three-electrode system with the voltage varying in the range of ±0.6 V. Figure 6 shows the surface potential, current as well as the COF of Cu over time in pure H_2_O lubricant. In the first 180 s of the running-in test (−180 s–0 s in the Figure 6), the surface of the lower friction pair Cu was in an open circuit voltage. After that, the three-electrode system was used to record the relationship among the current, surface potential, and time.

As shown in Figure 6A, the change of the current is basically the same as the change of surface potential, with the variation range of ±1 mA. Figure 6B presents the variation curve of friction coefficient of Cu plate vs. ZrO_2_ ball in pure H_2_O with time under different applied surface potentials. When the surface potential of the lower friction pair is positive, the COF decreases from 0.35 in the running-in period to 0.32. When the surface potential of the lower friction pair is negative, the COF increases to 0.45–0.50. At 60–120 s, 180–240 s, and 300–360 s, the existence of “twin peaks” is clearly observed in the curve of friction coefficient vs. time. The phenomena are considered to be caused by the formation and destruction of the tribofilm during the potential-controlled boundary lubrication process. In the three cycles, the COF changes well with the surface potential as well as the current.

**FIGURE 7:**
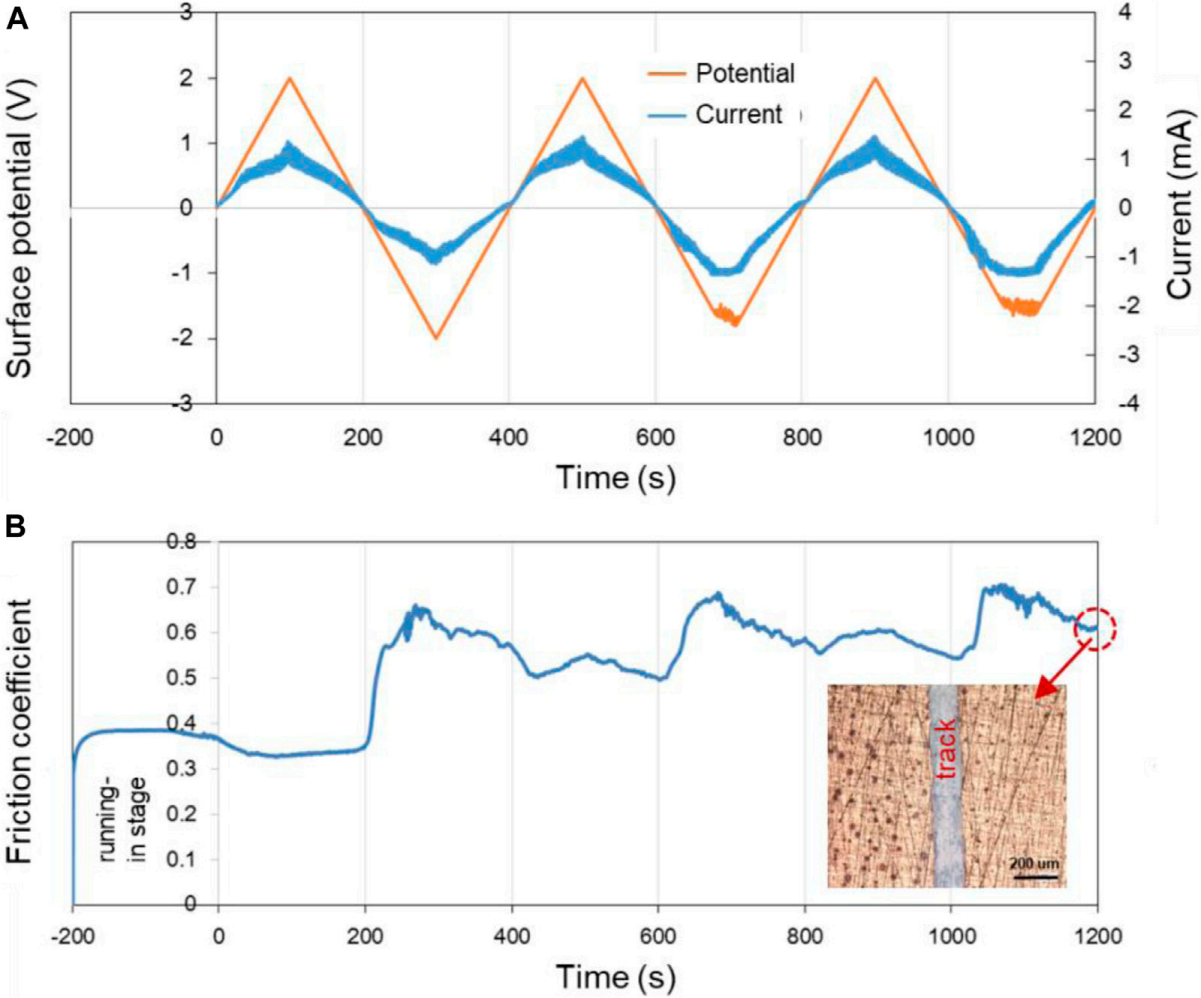
Potential-controlled friction results of the Cu plate vs. ZrO_2_ ball with pure H_2_O as lubricant and the Cu surface potential controlled among ±2.0 V. **(A)** The change of surface potential and current of Cu plate over time and **(B)** the variation curve of friction coefficient with time under the applied surface potentials in **(A)**. The insert in **(B)** shows the microscopic morphology near the track after the friction test.

According to Figure 6, when the applied potential goes from −0.6 V to +0.6 V, the COF changes reversibly with the surface potential of Cu plate. However, when other experimental parameters remain the same while the applied potential goes from −2.0 V to +2.0 V, the changes of the COF during the friction test become less reversible, as shown in Figure 7. The results reveal that in the former case, electrochemical reactions occur mainly in the contact area. While for the latter condition, the surface potential is high enough to cause the electrochemical reactions on the whole surface of the sample including contact area and noncontact area. It is obvious that the Cu surface suffered from corrosion as shown in the insert in Figure 7B. The OH^−^, H^+^, and metal ions formed by the electrolysis of water and corrosion of metals absolutely complicate the tribological behaviors of the system.

According to the above results, the potential-controlled boundary lubrication can be realized in pure H_2_O without any surfactant additives or charged nanoparticles. Comparing with the result in Figure 7, the reversibility of the experimental results shown in Figure 6 indicates that the mechanism maybe caused by the localized electrochemical reactions in the real contact area during the friction test instead of the traditional electrochemical reactions. In fact, when metals other than Cu, such as Fe, served as the lower friction pair, the COF at positive surface potential is lower than that at negative surface potential can also be obtained. According to the above model of triboelectrochemical reactions, the active control of friction in pure H_2_O is mainly influenced by changing the structure of the frictional oxidation film, which will be characterized and analyzed in the *Triboelectrochemical Products and Their Effects on Friction*.

### Potential-Controlled Boundary Lubrication in Pure Ester Oil

If the above phenomenon is general, the potential-controlled boundary lubrication effect should also occur in the case of the other pure liquids as lubricants. According to the achieved results, the active control of boundary lubricant by applied surface potentials might be related with the oxidation of the metal in the contact area. Therefore, the content of oxygen atoms in lubricants needs to be considered. Based on the above analysis, a kind of ester organic liquid, PC, was used as the model lubricant in this section, to comparing with the result of pure water. For one reason, ester oils are widely used in gears, jet engines, compressors, and other mechanical parts because of their high viscosity index and good lubrication effect. For another reason, the content of oxygen element in ester oils is relatively high and their molecules are generally polar, so it is predicted that the potential-controlled boundary lubrication effect in esters may be relatively significant.

**FIGURE 8:**
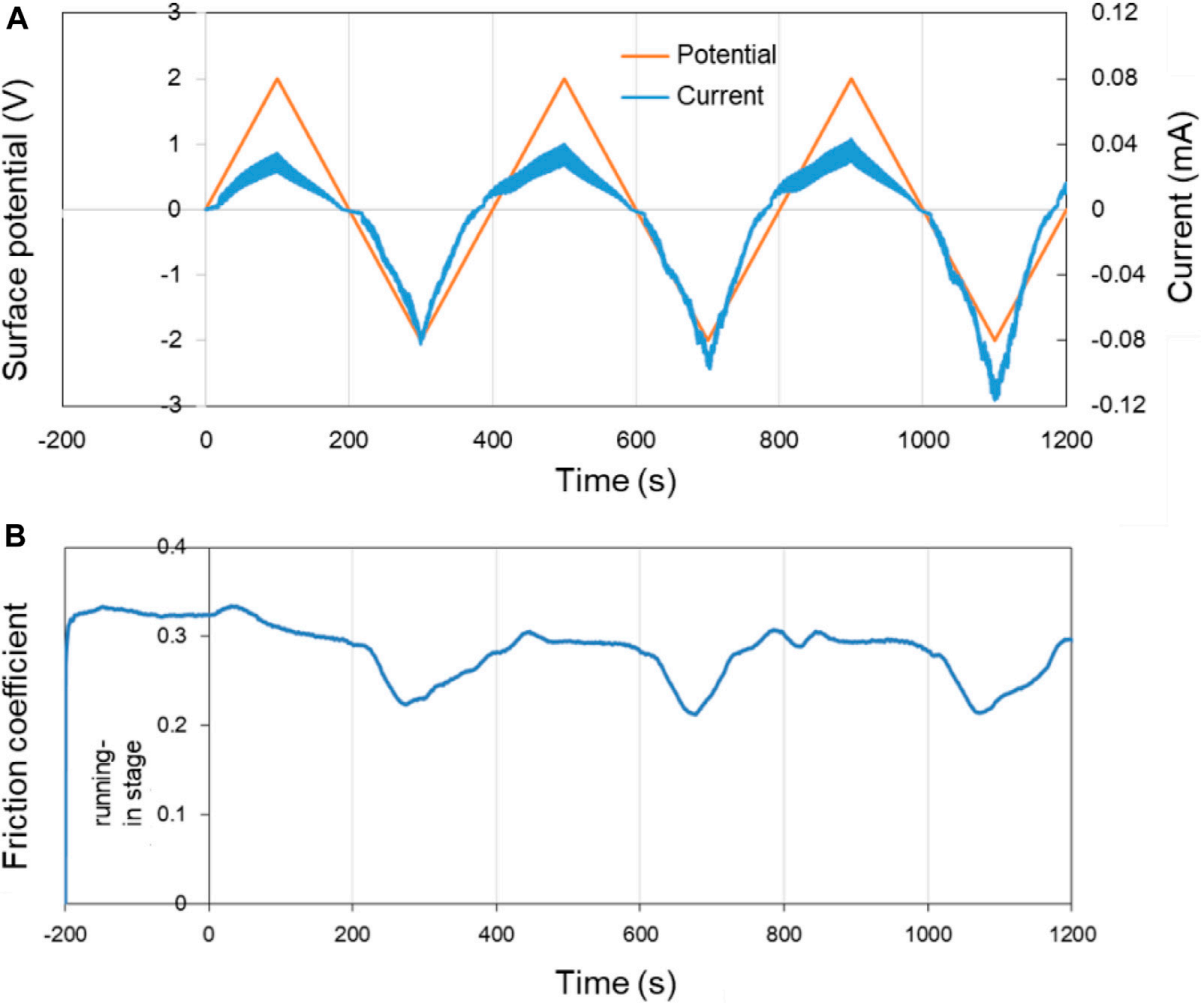
Potential-controlled friction results of the Cu plate vs. ZrO_2_ ball with pure PC as lubricant and the Cu surface potential among ±2.0 V. **(A)** The change of surface potential and current of Cu plate over time and **(B)** the variation curve of friction coefficient with time under the applied surface potentials in **(A)**.

Figure 8 shows the surface potential, current as well as the COF of Cu over time in pure PC lubricant. The surface potential of the Cu plate was controlled within ±2.0 V by using the three-electrode system. In the first 200 s of the running-in test (−200 to 0 s in the Figure 8), the surface of the lower friction pair Cu was in an open circuit voltage.

In three cycles of the surface potential, the change of current is consistent with the change of the surface potential. While with the increase of the time, the current at the same potential tends to increase. For example, although the surface potentials are all about −2.0 V at 300, 700, and 1,100 s, the current values are about −0.08, −0.10, and −0.12 mA, respectively. This phenomenon is considered to be related with the activation effect of friction. The fresh metal within the track of the Cu plate is exposed during the friction test, causing the contact resistance of the system reduced and the current increased under a certain surface potential. Figure 8B shows the COF of Cu plate over time under the control of the surface potential in Figure 8A. Differing from the result in Figure 7, the COF of Cu plate vs. ZrO_2_ ball decrease from 0.30 to ∼0.22 in PC lubrication when the surface potential of Cu is negative, and the experiment shows good repeatability.

For another ester oil, DES with relative weak molecular polarity working as lubricant, the tendency of COF to decrease can still be detected when negative surface potential of −2 or −4 V is applied on the Cu plate. While the COF changes with the surface potential, amplitude becomes smaller, comparing with the case of PC working as the lubricant. It is obvious that the type of liquid also has a great impact for the potential-controlled boundary lubrication behavior of pure liquid, in addition to voltage or surface potential. Because the polarity and functional groups for the different liquid molecules play an important role in the metal oxidation.

### Triboelectrochemical Products and Their Effects on Friction

The above friction results show that the effect of the potential-controlled boundary lubrication in pure H_2_O or ester lubricants is different. The COF of Cu plate vs. ZrO_2_ ball decreases when the surface potential of the Cu is positive in pure H_2_O lubricant. In pure PC lubricant, however, the COF decreases when the surface potential is negative with other experimental conditions unchanged. According to the triboelectrochemistry model and the definition of boundary lubrication (Wen and Huang, 2017), the active control of friction in pure liquid is considered to be caused by changing the formation of the oxidation film in friction contact area. In order to verify this analysis, the tribological experiments under a constant surface potential of Cu plate were carried out with different liquids as lubricants.

**FIGURE 9:**
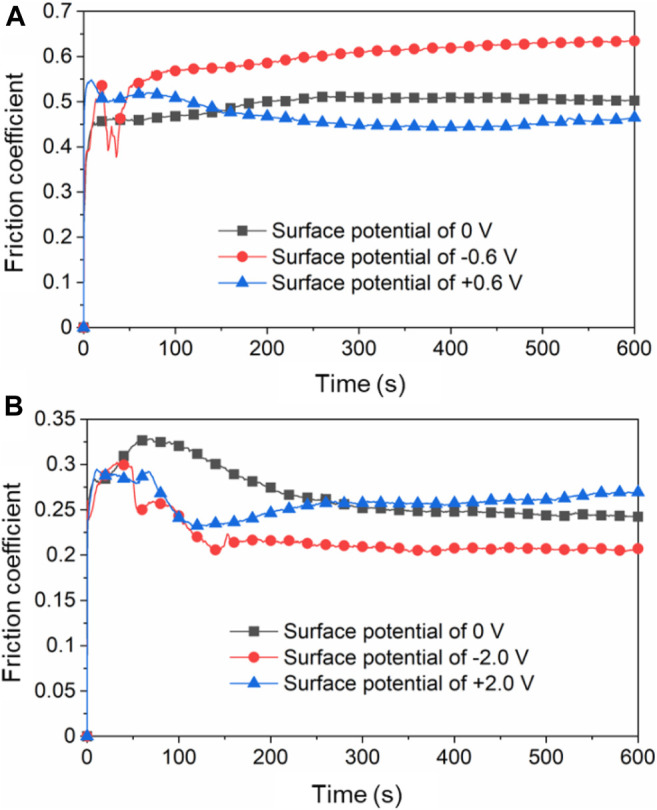
Friction coefficients of Cu plate vs. ZrO_2_ ball over time in **(A)** H_2_O as lubricant and **(B)** PC as lubricant under different surface potentials of Cu plate.

Figure 9 shows the COF over time in different lubricant under certain applied surface potentials. According to Figure 9A, when the surface potential of Cu plate is 0 V, the COF of the plate vs. ZrO_2_ ball is around 0.50 with pure H_2_O as the lubricant. The COF increases to around 0.60 when the surface potential is −0.6 V and decreased to 0.42 when +0.6 V is applied during the friction tests. The above results are basically consistent with that in Figure 6 when the surface potential of the lower friction pair changed continuously. Figure 9B presents the COF of Cu plate vs. ZrO_2_ ball over time with pure PC as the lubricant. When the surface potential is 0 or +2.0 V, the COF is around 0.25. When the surface potential is −2.0 V, the COF decreases to 0.20.

**FIGURE 10:**
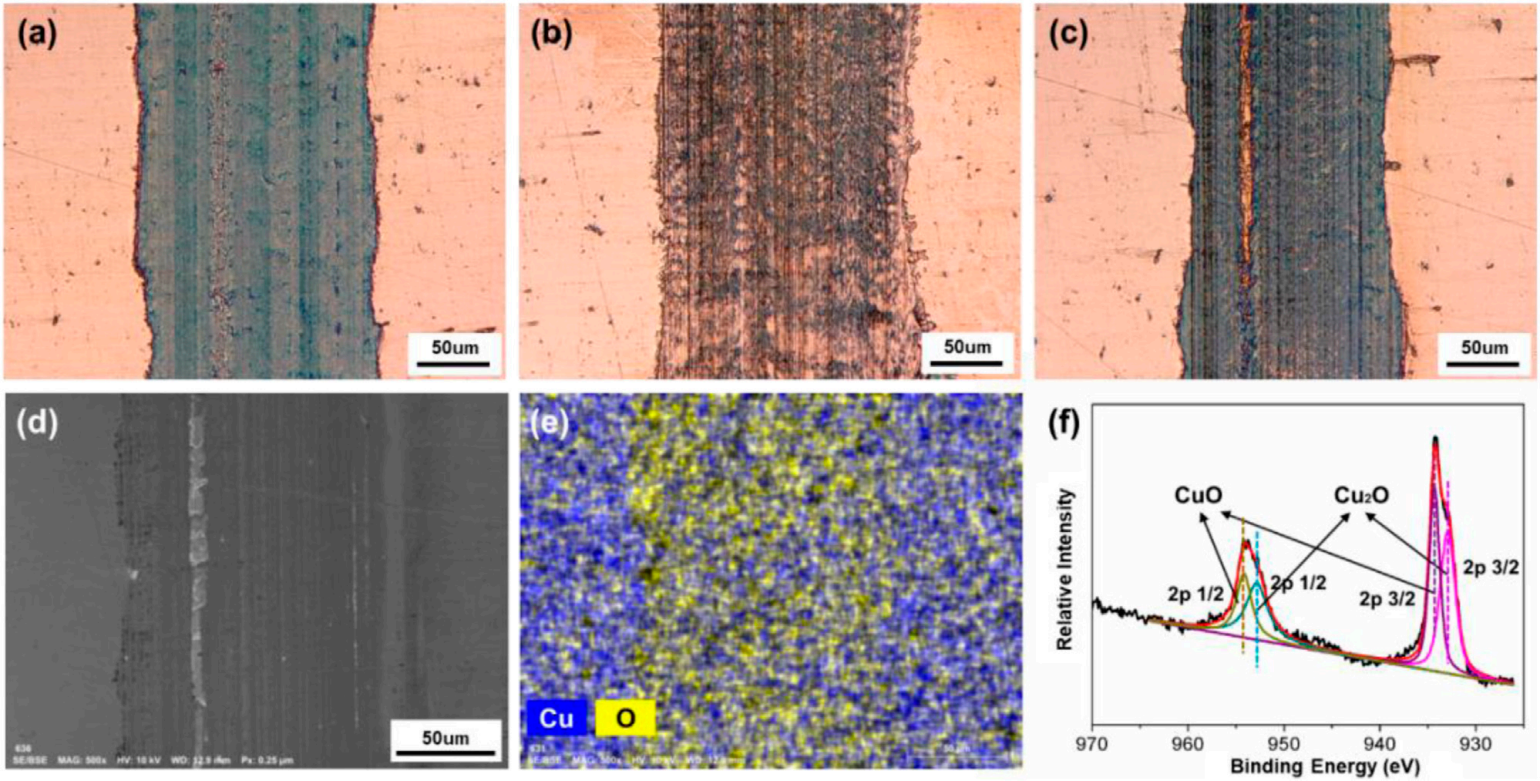
Surface morphologies and compositions of the tracks on the Cu plates produced under different friction conditions. Microphotographs of the tracks obtained under different applied surface potentials: **(A)** 0 V, **(B)** −0.6 V, and **(C)** +0.6 V. **(D)** SEM morphology, **(E)** EDS elemental map, and **(F)** XPS spectrum of Cu 2p of the tribofilms of the lower friction pair obtained under the positive surface potential of +0.6 V.

After the friction test, the surface morphologies and elemental compositions on the tracks obtained under different friction conditions were characterized and presented. In Figure 10, all of the three tracks are obtained on Cu plates after the 10 min of friction test with ZrO_2_ ball as upper friction pair and pure H_2_O as lubricant. When the surface potential is 0 V or +0.6 V, the track on the surface of Cu plate is dark brown and relatively uniform, with the width of 163 or 138 μm, as shown in Figures 10A,B. When the surface potential is −0.6 V, the color of track is close to that of the area without friction, and its width is about 176 μm, as shown in Figure 10C. Furthermore, SEM, EDS, and XPS were used to analyze the elements and components of the track obtained under the condition when +0.6 V of the surface potential on the Cu plate was applied. According to Figures 10D,E there are Cu and O elements observed within the track. The O element is uniformly distributed over the track. Figure 10F shows the XPS diagram of Cu 2p on the track. The Cu 2p peaks located at 954.18 and 934.34 eV are attributed to CuO. The difference between the two peaks is 19.84 eV, similar as the previously reported value of 20.0 eV (Vasquez, 1998; Barreca et al., 2007). The Cu 2p peaks located at 952.85 and 932.95 eV are attributed to Cu_2_O (Barreca et al., 2007). The above result indicates that CuO and Cu_2_O are present within the track of the lower friction pair obtained under positive surface potential of Cu plate.

Combining with friction test results in Figures 6, 9A, the observation of the tribofilm shown in Figure 10 involves that for the case of the condition with H_2_O used as lubricant, a continuous oxide film on the surface of Cu formed during the friction test can reduce the COF when positive surface potential is applied. However, when the surface potential is negative, the formation of the oxide film is inhibited in the contact area, causing a relative high COF. Moreover, although under the effect of surface potentials, no obvious reactions occur in the rest area except on the tracks in Figures 10B,C. The results show that the noncontact area is within the potential window, while the contact area is in the regime of tribolelectrochemical reaction.

**FIGURE 11:**
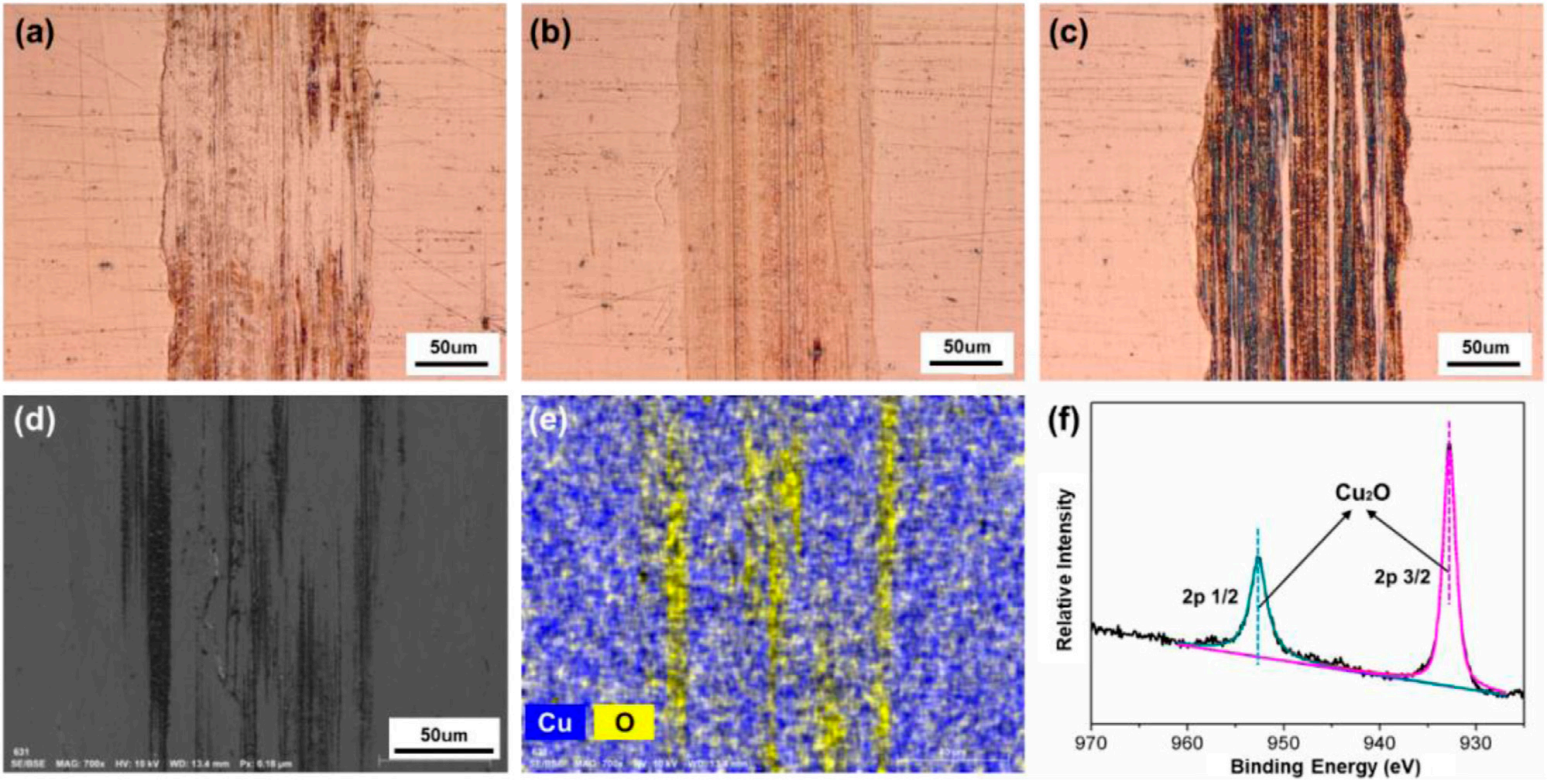
Surface morphologies and compositions of the tracks on the Cu plates produced under different friction conditions with pure PC as lubricant. Microphotographs of the tracks obtained under different applied surface potentials: **(A)** 0 V, **(B)** −2.0 V, and **(C)** +2.0 V. **(D)** SEM morphology, **(E)** EDS elemental map, and **(F)** XPS spectrum of Cu 2p of the tribofilms of the lower friction pair obtained under a positive surface potential of +2.0 V.

**FIGURE 12:**
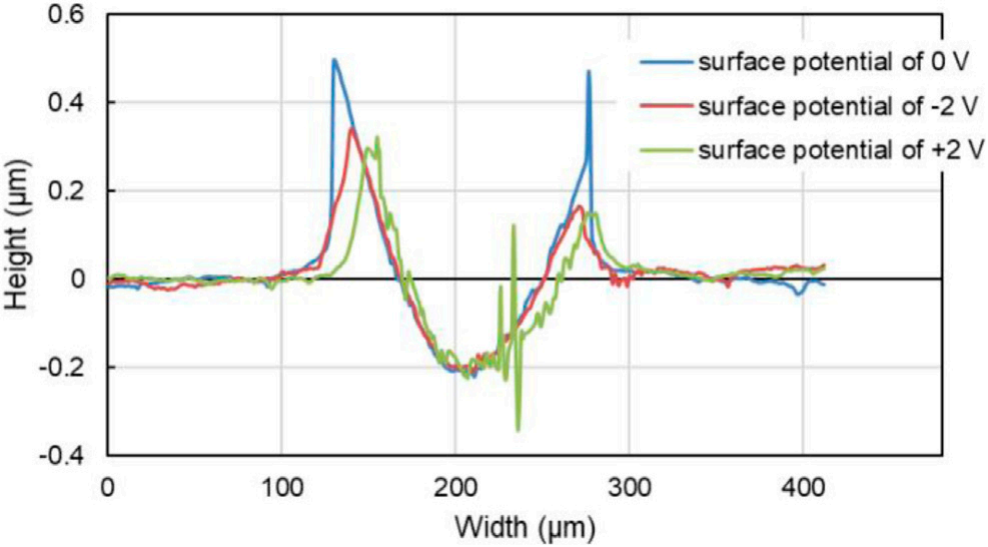
Transverse profile of the tracks on the Cu plates with different surface potential during the friction test of 10 min with pure PC as the lubricant.

In addition, the tracks of the lower friction pair after friction testing with PC as lubricant under different surface potentials were also characterized and shown in Figure 11. When the surface potential is 0 or −2.0 V, as shown in Figures 11A,B, the color of the tracks is almost the same with the untested area, and the width of the tracks is 177 μm or 164 μm, respectively. When the surface potential is +2.0 V, the track of the lower friction pair is dark brown and shows the obvious plowing phenomenon, with the width of 176 μm. Moreover, Figure 12 shows that when the surface potential of the Cu plate is +2.0 V, the profile curve of the track obtained with the surface potential of +2.0 V is the roughest comparing with the other two conditions.

Figures 11D–F are the results of microstructure and elements distribution on the track obtained under the positive potential test by SEM, EDS, and XPS, respectively. According to Figures 11D,E, there are Cu and O elements observed within the track. However, comparing with Figure 10E, the distribution of O element is nonuniform and shows a significant strip enrichment, which is considered to be caused by the plowing effect during the friction test. Figure 11F shows the XPS spectrum of the sample obtained under the positive surface potential of +2.0 V of the Cu plate. The XPS peaks of Cu 2p 1/2 and 2p 3/2 at 952.52 and 932.78 eV correspond to Cu_2_O, indicating that the process of the metal oxidation is slower than the case when pure H_2_O served as lubricant (Figure 10F).

To further analyze the formation of the tribofilms obtained when H_2_O or PC served as lubricant, the chemical properties of the lubricant should be discussed because the tribofilm is mainly formed by oxidation reaction between the metal and the oxygen in lubricant molecules. Comparing with H_2_O, the oxygen content as well as the chemical activity of oxygen in PC molecule is relatively insufficient, so the formed tribofilm is not dense enough. Especially during friction test, the non-dense oxidation product is easy to break down under the effect of the external force. As a result, the oxidation product plays the role of hard abrasive particles (comparing with the substance of Cu) and increases the COF. Hence, in the case of PC as lubricant, when the surface potential is negative, the COF is relatively low and smooth without the influence of the abrasive particles, comparing with the condition under positive surface potential.

## Localized Oxidation of Metal Surface Based on the Triboelectrochemistry

The triboelectrochemistry model can not only be used to explain the above potential-controlled boundary lubrication phenomena in pure liquids but also provide some new ideas to use the phenomena. Based on the model, a novel method for oxidation coloring of the selected areas in metal surfaces is proposed. The design principle is that friction can change the activation energy for electrochemical reactions, so that the oxidation of metals can be controlled and occurred in the contact region rather than in the noncontact area. That is to say, when the applied potential is relatively low, electrochemical reactions do not occur on the whole surface of the sample. However, if a local area is under the actions of proper load and frictional force, the threshold surface potential needed for electrochemical reactions is reduced due to the reduced chemical potential as indicated in Eq. 20. Hence, the triboelectrochemical reactions occur, limited inside that contact area. We named it as friction-induced oxidation in Figure 1. Because of the different electrochemical reaction regions, the selective oxidation and coloration of metal surface can be realized.

**FIGURE 13:**
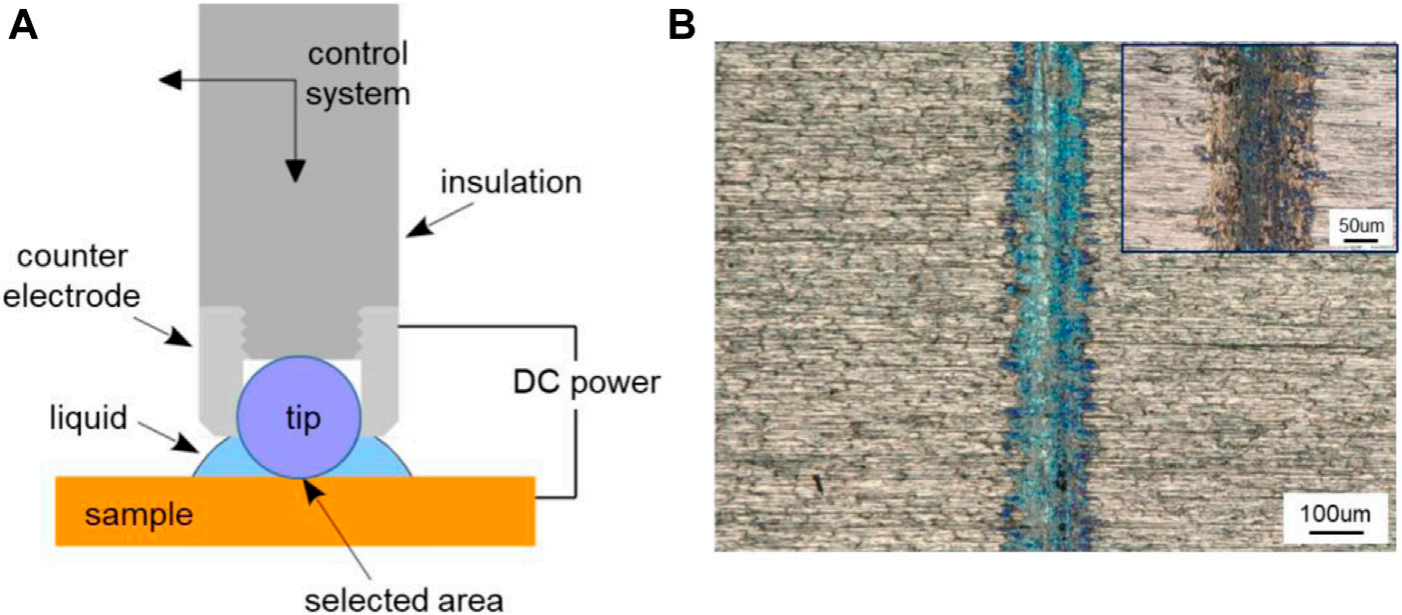
Localized oxidation coloring of metal surface based on triboelectrochemical reactions. **(A)** Schematic illustration of the device for localized oxidation coloring and **(B)** microscopic image of Ti surface coloring obtained with a positive potential of 1.5 V, and the image of the track obtained without applied potential as shown in the insert.

Figure 13A shows the schematic illustration of the device for localized oxidation coloring or electrolytic coloring. The ZrO_2_ ball with the diameter of 6.35 mm was used as the tip part of the device, and the pure Ti plate was used as the sample to be treated. Meanwhile, the Ti plate worked as the anode, a steel ball holder around the ZrO_2_ ball as the cathode, and the distance between them was about 1 mm. The load was about 2 N, the sliding speed was about 10 mm/s over a 5-mm track, and time was about 5 min. 0.5 mL PC liquid was added between the tip and the Ti plate. A direct current (DC) power supply was used in the experiment to provide the electric potential of 1.5 V. Figure 13B shows the effect diagram of Ti surface coloring, and the insert is the contrast picture without applied potential. Contrast with the two pictures in Figure 13B, an obvious difference can be obtained that when works as positive pole, the contact area of the Ti surface is blue, while there is no special color change for the track obtained in the absence of applied surface potentials. As we know, blue is a common color for the anodic oxidation of Ti metal and its alloys (Diamanti et al., 2008; Diamanti et al., 2011). However, the highlight in this work is that the oxidation coloring on the selected area of Ti surface is realized. By applying a reverse voltage, the oxidation coloring effect can be removed. This method is expected to have broad applications in the fields of micro-nano machining, information records, surface finishing, and so on.

## Conclusion

Focused on the solid–liquid interfaces under the coupling of multi-physics, a chemical potential equation for modeling triboelectrochemical reactions has been proposed (as shown in Eq. 20). It emphasizes the difference in electrochemical reactions between the friction contact area and noncontact area. The triboelectrochemical model can be intuitively applied to explain the observed phenomena of potential-controlled boundary lubrication for pure liquids and the formation of the triboelectrochemical products. Most importantly, a novel method for oxidation coloring of the selected areas in metal surfaces is proposed based on the model. Together with the adsorption and desorption model of lubricant additives, the triboelectrochemical reaction model can well explain the phenomena of potential-controlled boundary lubrication in different lubrication systems and also provide a theoretical basis for many solid–liquid interface processes under the effects of electromechanical coupling.

The proposed chemical potential equation still needs to be further discussed in the future and how to define and measure activation volumes may be the most challenging part. In addition, more applications on the triboelectrochemical reactions have yet to be developed.

## Data Availability

The raw data supporting the conclusion of this article will be made available by the authors, without undue reservation.

## Nomenclature

μ: chemical potential
$K^{\theta }$: dimensionless equilibrium constant of the chemical equilibrium
G: gibbs free energy
$\Delta_{r}G_{m}^{\theta }$: standard molar Gibbs free energy
$v_{B}$: stoichiometric number of component B in a reaction
R: gas constant
T: temperature
Δφ: change of electrode potential
$E_{a}^{0}$: activation energies of anodic reactions
$E_{c}^{0}$: activation energies of cathodic reactions
α: transfer coefficient of applied potential alters activation energy for reduction
β: transfer coefficient of applied potential alters activation energy for oxidation
*F*: faraday’s constant
*z*: reactive charge number
V(r): morse potential function
r: length of the bond among large molecules
$r_{0}$: equilibrium separation of the atoms
D: dissociation energy
k: force constant of the bond in the neighborhood of the equilibrium separation
f: tension
Δx: shift in position for the reactant A and product B
$k_{B}$: boltzmann constant
$K_{eq}\left(f\right)$: equilibrium constant with the external force
*P*: pressure
τ: shear stress
*Q*: activation energy to initiate sliding
*Ω*: pressure activation volume
ϕ: shear activation volume
$\mu^{\theta }$: chemical potential of a mole of substance A at standard pressure (101.325 kPa)
a: effective concentration of substance A, also named as activity
$\mu_{E}$: electrochemical potential
$a_{E}$: electrochemical activity
φ: potential of a system
$\mu_{P}$: mechanochemical potential
V: volume
$\mu_{f}$: tribochemical potential
$F_{P}$: pressure force
$F_{\tau }$: shear force
$l_{⊥}$: displacement in the direction of pressure force: displacement in the direction of shear force
$l_{⊥}$: displacement in the direction of pressure force: displacement in the direction of shear force
$a_{f}$: tribochemical activity
$\mu_{f\&E}$: triboelectrochemical potential
$a_{f\&E}$: triboelectrochemical activity
*a*_*eq*_: activity at equilibrium
φ_eq_: equilibrium potential in the absence of external force
${\varphi^{\prime }}_{eq}$: equilibrium potential under the action of a certain force
$\Delta \varphi^{\prime }$: change of the equilibrium potential under the action of a certain force
